# Power Efficient Random Access for Massive NB-IoT Connectivity

**DOI:** 10.3390/s19224944

**Published:** 2019-11-13

**Authors:** Mamta Agiwal, Mukesh Kumar Maheshwari, Hu Jin

**Affiliations:** 1 Department of Electrical Engineering, Sejong University, Seoul 05006, Korea; mamta@sejong.ac.kr; 2 Department of Electrical Engineering, Bahria University, Karachi 75260, Pakistan; mukeshkumar.bukc@bahria.edu.pk; 3 Department of Electrical Engineering, Hanyang University, Ansan 15588, Korea

**Keywords:** NB-IoT, random access, power consumption, coverage enhancement, collision probability

## Abstract

Sensors enabled Internet of things (IoT) has become an integral part of the modern, digital and connected ecosystem. Narrowband IoT (NB-IoT) technology is one of its economical versions preferable when low power and resource limited sensors based applications are considered. One of the major characteristics of NB-IoT technology is its offer of reliable coverage enhancement (CE) which is achieved by repeating the transmission of signals. This repeated transmission of the same signal challenges power saving in low complexity NB-IoT devices. Additionally, the NB-IoT devices are expected to suffer from congestion due to simultaneous random access procedures (RAPs) from an enormous number of devices. Multiple RAP reattempts would further reduce the power saving in NB-IoT devices. We propose a novel power efficient RAP (PE-RAP) for reducing power consumption of NB-IoT devices in a highly congested environment. The existing RAP do not differentiate the failures due to poor channel conditions or due to collision. After the RAP failure either due to collision or poor channel, the devices can apply power ramping or can transit to a higher CE level with higher repetition configuration. In the proposed PE-RAP, the NB-IoT devices can re-ascertain the channel conditions after an RAP attempt failure such that the impediments due to poor channel are reduced. The power increments and repetition enhancements are applied only when necessary. We probabilistically obtain the chances of RAP reattempts. Subsequently, we evaluate the average power consumption by devices in different CE levels for different repetition configurations. We validate our analysis by simulation studies.

## 1. Introduction

**Figure 1:**
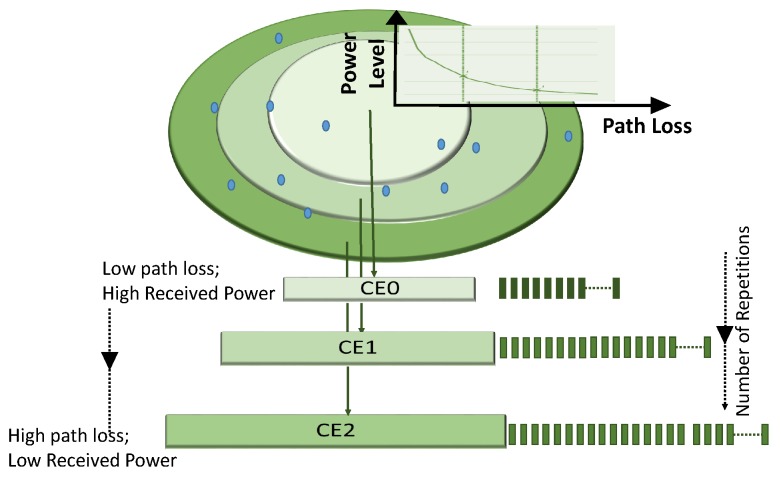
Coverage enhancement levels and repetitions.

The Internet of things (IoT) has been gaining popularity for its smart decision making capabilities using data acquired through several sensors. The narrowband Internet-of things (NB-IoT) is the standardized form of the IoT that is compatible with the legacy Long Term Evolution(LTE) mobile networks. It is especially attractive for low energy sensor based applications that require wireless data transmission using the backbone network. NB-IoT is imbued with features like low complexity, low cost, ubiquitous coverage, low data rate and low power computing. One of the important NB-IoT attributes is its offer of significant coverage extension beyond existing cellular technologies [1,2]. The coverage extension feature of NB-IoT technology is especially useful when sensors are located in remote or hard to reach areas. Reliable coverage enhancement is achieved by the repeated transmission of data and control signaling. Each transmission can be configured to repeat for a designated number of times in order to achieve higher success opportunities at the desired coverage level [3]. When path loss at the desired coverage is high, a greater number of repetitions can be configured. The network can configure up to three coverage enhancement (CE) levels (CE0, CE1, CE2) to serve User Equipments (UEs)with different path losses [4] as shown in Figure 1. The main impact of the different CE levels is that the message is to be repeated a different number of times. If the device resides in the CE level with lower path loss (level CE0 in Figure 1) then the number of times that the signal needs to be repeated is less. However, the number of configured repetitions are higher for the device in the CE level that manifests high path loss (level CE2 in Figure 1). This ensures that a good signal quality is received in all the configured CE levels.

Repeated transmission occupies more resource elements [5] leading to loss in spectral resources. Thus, several of the recent works on NB-IoT are focused on evaluation of the optimal number of repetitions. Not only the spectral resources, but the power consumption of the NB-IoT devices would also be affected by the increase in the number of repetitions. In light of distinctive NB-IoT requirements like the battery life of ten years, small form-factor battery, support for low-power and low complexity operation [1], it is not hard to visualize the importance of conservative methods for achieving the power efficiency objectives. In this paper, we specifically focus on manifesting power saving in the random access procedure (RAP). In RAP, the device that wishes to establish the connection with the base station randomly chooses and transmits a preamble. The legacy RAP was originally designed for a limited number of connections. However, the number of devices that attempt RAP in massive connectivity would be high. The vision of a connected IoT is based on the usage of a high number of low complexity wireless devices. As the technology advances, people would be using an increasingly broader range of services and applications [6]. The requirements for enormous connectivity would only become more acute in the future. In such a congested environment, the RAP would often fail due to frequent collisions of preambles [7]. The resulting multiple RAP reattempts would adversely affect the power saving of the NB-IoT devices. It is notable that each RAP reattempt in turn comprises of several repetitions of preamble transmission. Thus, we believe that it is important to address the power saving challenge when a congested RAP is considered together with the transmission repetition characteristics of NB-IoT devices.

If an RAP attempt by an NB-IoT device fails, then the device can first reattempt the RAP in the same CE level as the one with the preceding attempt; 3GPP defines the parameter ‘maxNumPreambleAttemptCE’ such that when the preamble transmission counter of the CE level becomes higher than this parameter, the CE level is increased [8]. Thus, after the configured number of reattempts in the initially selected CE level, the RAP reattempts are performed in the higher CE levels [9]. The number of repetitions and the power level are high in the higher CE level. Moreover, the RAP reattempts can also be performed by power ramping power, i.e., by increasing the transmission power (3GPP defines parameters (*‘powerRampingStep’* and optionally *‘powerRampingStepCE1’*)) [8,10]. In a massively connected NB-IoT environment where collisions are umpteen, the power ramping and sequential CE level upgradations would sharply degrade the performance of RAP in terms of power consumption. While it is admissible to increase power due to poor channel conditions, power ramping may not be necessary when failures occur due to collisions. Thus motivated, we propose a novel power efficient RAP (PE-RAP) for NB-IoT devices in congested environments. In PE-RAP, the devices do not indiscriminately perform power ramping nor do they unnecessarily move to the higher CE level for RAP reattempts. The devices have flexibility to re-select the CE level after failure in PE-RAP such that the channel conditions are re-ascertained at every RAP reattempt. More precisely, our contributions are as follows:

1. In PE-RAP, the devices reaffirm the channel conditions. Subsequently, the appropriate power level and repetitions are re-selected such that chance of failure due to poor channel are reduced. Higher CE levels are configured for transmission at higher power and thus power ramping becomes implicit.
2. Every RAP reattempt causing more power consumption can occur only after the collisions in PE-RAP. Thus, we also evaluate the collision probability of the devices.
3. The access class barring (ACB) mechanism is popular for congestion control in machine type communications. ACB probabilistically controls the preamble transmissions by the devices. We incorporate the ACB mechanism in our proposal since high connectivity is considered.
4. We analyze the average power consumption in the proposed PE-RAP and compare it to the existing NB-IoT RAP. Additionally, extensive simulations are carried out to validate the analytical results.

The rest of the paper is organized as follow. The NB-IoT physical random access channel (NPRACH) fundamentals and related work are delineated in Section 2 to highlight the effect of repetitions and collisions on NB-IoT devices. Section 3 presents the details of the proposed PE-RAP mechanism and its analytical modeling. The mathematical model for power consumption in the existing NB-IoT RAP is derived in Section 4. In Section 5, the numerical and simulation results are presented. Finally, Section 6 summarizes the conclusion.

## 2. Literature Survey Random Access in NB-IOT

Low cost, low power, reliable and robust applications are becoming popular for services like traffic surveillance, military sensing, industrial automation, manufacturing, environment monitoring, medical services and physical security [11,12]. NB-IoT has been specifically introduced to provide low-cost, low-power and wide-area cellular connectivity [1]. It is designed to operate at 180 kHz system bandwidth, that corresponds to one physical resource block (PRB) in the legacy LTE transmission [13]. Its design is based on existing LTE functionalities. However, the 180 kHz bandwidth presses for several simplifications and modifications over existing procedures [1]. To this regard, a novel NPRACH is designed for NB-IoT. In this section, we first delineate the novelty of NPRACH while highlighting the repetition mechanism. Subsequently, we present a concise review of existing literature on random access in NB-IoT.

### 2.1. NPRACH Fundamentals

**Figure 2:**
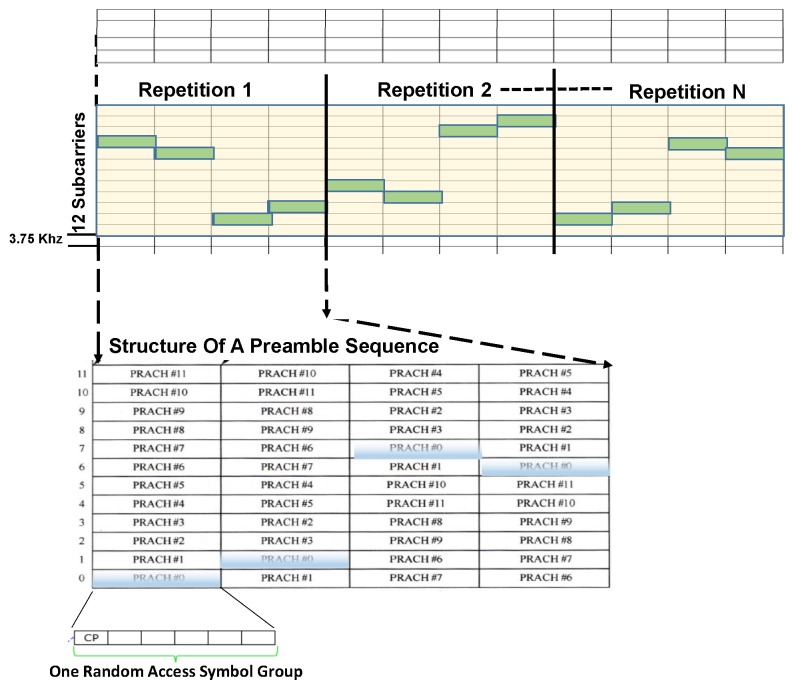
Novel narrowband Internet-of things (NB-IoT) physical random access channel (NPRACH) design for NB-IoT.

Similar to legacy LTE/LTE-A networks, RAP in NB-IoT is a four-step process [14]. However, unlike LTE in step one, an NB-IoT device starts RAP by transmitting a newly designed preamble in the NB-IoT physical random access channel (NPRACH) [15]. NPRACH is the time-frequency resource for the transmission of random access preambles in NB-IoT. While PRACH in LTE uses a bandwidth of 1.08 MHz, NB-IoT uplink bandwidth is limited to only 180 KHz, which is far less. Thus, NPRACH is focused on a newly designed preamble format such that each preamble consists of four symbol groups [14]. Thereupon, each symbol group comprises of five symbols and a cyclic prefix. The NB-IoT preamble transmission comprises of 4 symbol groups transmitted without gaps [3]. While each group uses a single subcarrier, it can hop across 12 subcarriers as shown in Figure 2 which facilitates uplink timing estimation at the eNodeB (eNB) [16]. The device can repeat the preamble transmission for up to 128 times in each RAP attempt. The exact number of repetitions is based on the CE level of the device [3,17]. Each repetition consists of symbol groups transmitted at four different subcarriers (within a band of 12 subcarriers) that follow a predefined frequency hopping pattern [18]. The preamble is uniquely identified by its hopping signature and therefore can be differentiated based on the choice of the initial subcarrier. In Figure 2, the hopping pattern of PRACH index #0 is highlighted in blue. With the subcarrier spacing of 3.75 KHz, the NPRACH is a set of 48 subcarriers on anchor carrier with a basic sub-carrier allocation unit of 12 sub-carriers [9]. Thus, 12,24,36 or 48 orthogonal preamble sequences are possible on an anchor carrier which are shared by up to three CE levels [19]. It is to be noted that one subcarrier can be assigned to only one CE level.

To initiate RAP, the device transmits its preamble using randomly chosen subcarriers mapped to its initially selected CE level. The device identifies its initial CE level by measuring reference signal received power (RSRP). Devices with the highest value of RSRP are in the lowest initial CE level (CE0), while the devices that measure the lowest RSRP are in the highest initial CE level (CE2). Each NPRACH resource configuration, corresponding to different CE levels, is characterized by its own parameters like periodicity, number of repetitions, starting time, frequency location and number of subcarriers [20]. The choice of the subcarrier for preamble transmission is random and independent of the choice made by other devices. If more than one devices from the same CE level select the same initial subcarrier in the same PRACH occasion, it results in a collision.

### 2.2. Related Work

Since its introduction in 3GPP Rel-13, NB-IoT has gained increasing research interest for its ability to address low complexity, high coverage, long device battery life and massive capacity objectives [1,2]. Performances and design considerations of NB-IoT, spanning over its evolutions, technologies and issues are summarized in recent works [1,2,21,22]. More specifically, the works on random access in NB-IoT are focused on either the detection of superimposed NPRACH preambles or the optimistic resource allocation [4,7,9,14,23,24,25,26].

In Ref. [7], the NB-IoT random access procedure is probabilistically modelled using Markov chain for the evaluation of system throughput in terms of number of devices, re-transmission number, packet generation rate and the length of the queue. Research works in [9,14] consider non-overlapping sub-carriers reserved by the eNB for the three different CE levels to derive success probability for devices located in the different levels. Authors in [9] present an analytical model to estimate success probability and delay in RAP while considering three CE levels of NB-IoT deployment. Harwahyu et al. in [23] propose a joint optimization technique under a target delay constraint. The work presents optimal configuration of NPRACH parameters in order to maximize the access success probability [23]. Access reservation protocol with partial preamble transmission is presented in [24]. The partitioning of preamble facilitates reuse of the same preamble sequence by several devices. While the proposal mitigates collision probability, it is accomplished by the degradation of detection performance [24]. A novel random access model that considers the NB-IoT traffic characteristics is presented in [25]. For Beta and uniform arrivals throughput and delay are analyzed. When multiple devices attempt RAP simultaneously, the eNB receives superimposed NPRACH preambles [4]. In such an environment detection of all users is a challenge and thus the work in [4] formulates a framework for multi user detection problem. The capacity gain is estimated in [26] while considering that massive number of NB-IoT devices access network with delayed back-off value. Trade off between reattempts of random access and repetition of preambles is investigated in [14]. The results show that even though the large repetition values increase the detection probability at physical layer, they do not necessarily mean higher success of RAP at MAC-layer [14]. It also highlights that the average energy consumption increases with the increase in number of repetitions. It is not only intuitive but also clear from recent works [14,27] that repetitions increases energy expenditure. Moreover, all the aforesaid works have highlighted the increase in collisions when NB-IoT devices perform access in massive connectivity. Thus we believe that is it important to analyse power consumption while devices perform RAP in high contention and high repetition NB-IoT environment. Table 1 summarises the related work section.

## 3. Collision—CE Level Based RAP (PE-RAP)

**Figure 3:**
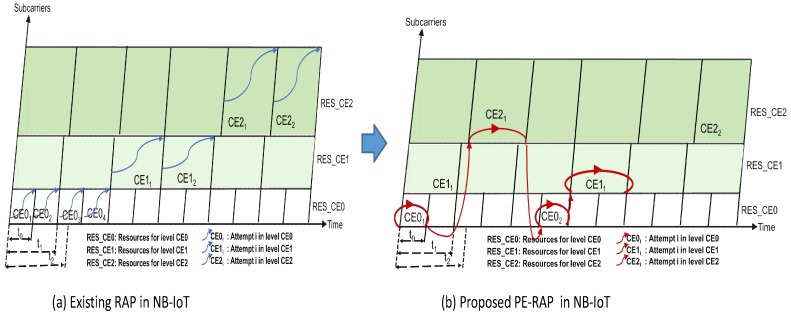
(**a**) Existing RAP in NB-IoT; (**b**) Proposed PE-RAP Mechanism.

The network configures CE levels and broadcasts the list of power thresholds for the received reference signals of the CE levels in the cell [3]. The device measures RSRP for the selection of its CE level. Subsequently, it randomly selects the preamble from amongst the ones mapped to the chosen CE level; 3GPP defines that each PRACH resource contains a set of subcarriers and every subcarrier corresponds to a random access preamble [8]. The number of preambles allocated to each CE level are limited and are configured by the network. In a massively connected IoT where enormous number of devices may perform simultaneous random access, more than one device may choose the same preamble resulting in an RAP failure. In the existing NB-IoT system shown in Figure 3a, the device on an RAP failure first reattempts in its initially chosen CE level. The device keep trying RAP reattempts in its selected CE level until the maximum number of configured attempts in this CE level are executed [9]. Subsequently, if the failure persists, the RAP restarts in the next higher CE level [8]. Several RAP reattempts are allowed until the device preempts all possible trials up to the highest CE level. We propose that in PE-RAP, the device measures RSRP after every failure and is able to re-select the CE level. Thus, on an RAP failure, the device do not necessarily move to the higher CE level in a sequential order. In  PE-RAP, the device that fails an RAP attempt has the flexibility to transit to any of the CE levels as shown in Figure 3b. Moreover, in PE-RAP the NB-IoT device is not required to perform power ramping when reattempting an RAP in the same CE level. Higher CE levels are configured for transmission at higher power. When the device selects higher CE level for its RAP reattempt then the power ramping becomes implicit. By enabling the re-selection of the CE level on an RAP failure, PE-RAP enables the correct selection of the number of repetitions and the power level such that they are neither unnecessarily high nor inadequately low. This is because different CE levels are configured with different power level and different number of repetitions. The proposed PE-RAP is presented in Algorithm 1. It has the following key components:

**Algorithm 1** PE-RAP
1: **for** 1: T trials **do**2: Input: $q_{0},\;q_{1},\;q_{2},\;R_{0},\;R_{1},\;R_{2},\;N,M_{s}\;\&\;\rho$3: Output: Number of attempts in each CE level and $P_{avg}$4: Devices select either CE0 or CE1 or CE2 with the probability $q_{0}$ or $q_{1}$ or $q_{2}$, respectively5: Let $n_{1}$ devices select CE0 out of *N* devices6: $n_{1t}=n_{1}+$ New arrival + Devices that did not attempted for RAP during previous $t_{i}-1$ slot7: $n_{1t}$ Device selects a random number between [0,1]8: **if** Selected number is > Optimal ACB factor $\rho_{*}$, **then**9:  Remaining $m_{k}$ devices out of $n_{1t}$ devices attempt for RAP during this $t_{i}$10:  Pr(mk∣n1t)=n1tmkρ*mk(1−ρ*)n1t−mk
11:  i=i+1 (Count attempt in CE0)12:  **if** Two device selects same preamble from $M_{s}$, **then**13:   It is considered as collision and collision probability is $P_{r}\left(C\right)$14:   The collision results in random access attempt failure15:   Go to step 416:  **else**17:   Device is successful18:  **end if**19: **else**20:  Wait for $t_{i}+1$ and go to step 421: **end if**22: Similar Process is Repeated for CE1, and CE2 to obtain *j* and n−i−j23: Pavg[n]=∑i=0n∑j=0n−inin−ij(iR0P0+jR1P1+(n−i−j)R2P2)q0i(1−q0−q2)j(q2)n−i−j
24: $P_{avg}=\sum_{n=1}^{\infty }P_{avg}\left[n\right]P_{r}{\left(C\right)}^{n-1}\left(1-P_{r}\left(C\right)\right)$
25:
**end for**

- Inputs $R_{0},R_{1},R_{2},$
  It is clear from the 3GPP report on random access in NB-IoT that the PRACH configuration includes the parameter $N_{rep}^{NPRACH}$. It gives number of NPRACH repetitions per RAP attempt [18]. It is not a fixed value and would depend upon the network configurations. For PE-RAP, we consider variables $R_{0},R_{1}$ and $R_{2},$ respectively, to express the number of repetitions in CE0, CE1 and CE2. For a reliable transmission at high coupling loss more number of repetitions can be configured [28]. In the succeeding performance analysis section, we give different values to $R_{0},R_{1}$ and $R_{2},$ such that ($R_{0}<R_{1}<R_{2}<128$) and observe their effect on the power consumption.
- Inputs $q_{0}$, $q_{1}$ and $q_{2}$
  As there are three CE levels, $q_{0}$, $q_{1}$ and $q_{2}$, respectively, represent the probabilities of channel conditions for CE0, CE1 and CE2 such that $q_{0}+q_{1}+q_{2}=1$. The device that fails RAP can re-select any of the CE levels for the RAP reattempt based on the probabilities $q_{0},q_{1}$ and $q_{2}$. The device measures RSRP to select the CE level. Since in PE-RAP we consider that the CE level is re-selected at failure, it can be accomplished by the RSRP measurements in the real world scenario. Since our proposal is aimed at obtaining the average power consumption, the specific methodology for the measurement of RSRP is not in the scope. Instead, we use probabilities $q_{0}$, $q_{1}$ and $q_{2}$ to emulate that the conditions where the device can be in any of the CE levels after the collision. The probability that the device selects CE0 for an RAP reattempt is $q_{0}$. Similarly, the device can select CE1 or CE2 with probability $q_{1}$ or $q_{2}$ respectively for its RAP reattempt. In the performance analysis we vary $q_{0}$, $q_{1}$ and $q_{2}$ to understand the effect of CE level variations. The channel variation over time is possible since each RAP attempt comprises of several repetitions over which the channel may change. Moreover, the device has to wait for the next PRACH occasion before it can reattempt an RAP.
- RAP and preamble selection
  To initiate an RAP, an active NB-IoT device selects the preamble randomly from amongst the ones that are mapped to its identified CE level. In step 5 of the algorithm, if the device is in CE0, it selects the preambles configured for CE0. The device competes for preambles with other newly arrived devices as well as the backlogged devices from the previous RAP attempts (highlighted in Step 6). For new arrival we consider Beta distribution. The simultaneous access by massive number of NB-IoT devices would result in congestion.
- Massive Connectivity and Collisions
  To improve the access quality-of-service in machine type traffic, the access class barring (ACB) scheme is widely adopted [29]. In steps 7 to 11, the devices perform an ACB check. Two or more NB-IoT devices that pass the ACB check can send a random access attempt of RAP by selecting the same preamble. If the preamble is the same, it would result in collision.
- Collision Probability and Access Attempt Failure
  According to 3GPP, the choice of the number of repetitions in each CE level is targeted to achieve 99% detection probability of the preamble [14,30]. The CE level is re-ascertained at each reattempt in PE-RAP. This ensures that the correct number of repetitions is selected such that the impediments due to poor channel become negligible. In other words we can say that the chances of RAP failure due to collision are much higher than due to preamble not being detected in poor channel conditions. Thus, in the PE-RAP algorithm it is assumed that the reattempt can occur only after the collision. We evaluate the collision probability $P_{r}\left(C\right)$ (step 13). The collision probability is calculated in the subsequent subsection and is used to obtain the average power consumed by the device unconditioned to the number of attempts (step 24). Step 15 shows that the device goes back to step 4 for CE level re-selection if the collision occurs.
- Number of attempts and average power evaluations
  In a massively connected environment, the RAP may fail often due to collision events. After the collision the device should perform an RAP reattempt and each reattempt is accomplished by several repetitions. To evaluate the average power consumption PE-RAP, we count the number of attempts that the devices make in each of the CE levels (Step 11 for CE0). The devices that are successful are removed from the system (step 17). Finally, from steps 23 and 24 we can obtain the average power in PE-RAP over n trials and unconditioned to n, respectively.
- Power Ramping
  As clear from the algorithm, power ramping is not applied. If the device after collision selects the same or a lower CE level, then power ramping would cause unnecessary wastage in low power NB-IoT devices. Thus, In PE-RAP, the device has the option to reattempt RAP in the same CE level at the same power. It can also transit back from the higher CE level to a lower CE level if the channel improves in the subsequent attempt. Since lower CE levels are configured for transmission at lower power levels, the power saving is substantial. Moreover, if the channel deteriorates while an RAP attempt is made, the device has the feasibility of selection of higher CE levels for its reattempt. In case of a reattempt at the higher CE level, the preamble transmission is performed at higher power. Thus, power ramping becomes implicit and is not explicitly included in the algorithm.

The aim of PE-RAP is to highlight the power consumption of NB-IoT devices due to (i) repetitions per transmission and (ii) massive connectivity on the random access procedure. In the next subsection we evaluate the average power consumption of NB-IoT devices over different repetitions and channel probabilities. In PE-RAP, a reattempt can occur only after the device fails an RAP attempt due to collision. Thus, we base our analysis on collision probability and the probability of the device belonging to a particular CE level. The list of parameters used in the analysis is delineated in Table 2.

### 3.1. Average Power Consumption in PE-RAP

As there are three CE levels, $q_{0}$, $q_{1}$ and $q_{2}$, respectively, represent the probabilities of channel conditions for CE0, CE1 and CE2 such that $q_{0}+q_{1}+q_{2}=1$. The device that fails RAP can re-select any of the CE levels for the RAP reattempt based on the probabilities $q_{0},q_{1}$ and $q_{2}$. Let the device succeed in its RAP after *n* number of reattempts where, unlike existing system, each RAP trial is independent of the previous attempt. $P_{r}\left(C\right)$ represents the collision probability such that a collision results in a failed RAP attempt. Since the choice of preamble by any active NB-IoT device is random and independent of the other devices, more than one active device may end up selecting the same preamble which would result in the collision. Then $P_{r}\left\{n=1\right\}=\left(1-P_{r}\left(C\right)\right)$ gives the probability that the RAP is successful in one attempt. Thus, the probability of an RAP being a success in *n* attempts can be expressed as

(1) $$\begin{array}{cc} \; & P_{r}\left\{n\right\}=P_{r}{\left(C\right)}^{n-1}\left(1-P_{r}\left(C\right)\right) \end{array}$$

Of total *n* RAP reattempts to achieve success, the device could have performed any of its RAP attempts in any of the CE levels. Unlike the existing NB-IoT RAP the order and count of reattempts in different CE levels is not crucial in the proposed system. Let *i* be the number of RAP reattempts that the device performs in CE0 and *j* be the RAP reattempts in CE1. The remaining out of *n* are performed in CE2. In each CE level the number of repetitions are different. The required preamble repetitions are small in level CE0 and are greater for level CE2. If $R_{0}$, $R_{1}$ and $R_{2}$, respectively, delineate the number of repetitions in CE0, CE1 and CE2, then $R_{0}<R_{1}<R_{2}$. A high number of repetitions translates into more power consumption. The power consumption is different in different CE levels. If $P_{0},P_{1}$ and $P_{2}$, respectively, delineate the power levels for each repetition in CE0, CE1 and CE2, then the average power consumed by the device over *n* reattempts can be expressed as

(2) Pavg[n]=∑i=0n∑j=0n−inin−ij(iR0P0+jR1P1+(n−i−j)R2P2)q0i(1−q0−q2)j(q2)n−i−j

Subsequently, we can obtain the average power consumed by the device unconditioned on *n* as

(3) $$\begin{array}{cc} P_{avg} & =\sum_{n=1}^{\infty }P_{avg}\left[n\right]P_{r}\left\{n\right\}=\sum_{n=1}^{\infty }P_{avg}\left[n\right]P_{r}{\left(C\right)}^{n-1}\left(1-P_{r}\left(C\right)\right) \end{array}$$

The equation clearly shows the importance of collision probability in evaluating consumed power in PE-RAP. Thus, we elaborate on the evaluation of $P_{r}\left(C\right)$ in the next subsection. Since our system model considers a congested environment, we also incorporate the ACB factor, popular for congestion control, in our analysis.

### 3.2. ACB Factor and Collision Probability

Let there be ${}^{\prime }N^{\prime }$ NB-IoT devices spread over all the three CE levels in a cell; 3GPP proposes an ACB mechanism such that the ACB factor ρ∈[0,1] is broadcasted for the congestion control of RAP [29]. The device randomly chooses a value between [0,1] and attempts RAP only if it is greater than the broadcasted value of ρ. We consider that $m_{k}$ out of *N* devices pass the ACB check and attempt RAP when there are $M_{s}$ subcarriers configured by the network for the RAP at the given point. Then, $P_{r}\left(m_{k}|N\right)$ gives the probability that $m_{k}$ out of *N* NB-IoT devices in the cell attempt the RAP, by transmitting with the optimal transmission probability $\rho_{*}$ where $\rho_{*}$ maximizes the average number of successes [29]. $P_{r}\left(m_{k}|N\right)$ can be expressed as

(4) Pr(mk∣N)=Nmkρ*mk(1−ρ*)N−mk

$P_{r}\left(M_{s}\right)$ gives the probability that the subcarrier for preamble transmission selected by an active device is also selected by another active device and it can be expressed as

(5) $$\begin{array}{cc} P_{r}\left(M_{s}\right) & =\left(1-{\left(1-1/M_{s}\right)}^{m_{k}}\right) \end{array}$$

The NB-IoT device in PE-RAP reattempts to access the network only after a collision. The collision can occur if any other device, out of N−1 remaining devices, selects the same subcarrier for preamble transmission. The collision probability therefore can be obtained as

(6) Pr(C)=∑mk=1N−1Pr(mk∣N)Pr(Ms)Pr(C)=∑mk=1N−1N−1mkρ*mk(1−ρ*)N−mk−11−(1−1/Ms)mk

From work in [31], we can get the optimal value of $\rho_{*}$ that maximizes the probability of transmission as min($1,\frac{M_{i}}{m_{i}}$). Using the optimal value of $\rho_{*}$, Equation (6) can be rewritten as in Equation (7) which can be elaborated as delineated in Equation (8).

(7) Pr(C)=∑mk=1N−1N−1mkMsNmk1−MsNN−mk−11−(1−1/Ms)mk

(8) Pr(C)=∑mk=1N−1N−1mkMsNmk1−MsNN−mk−1︸Θ1(Pr(C))−∑mk=1N−1N−1mkMs−1Nmk1−MsNN−mk−1︸Θ2(Pr(C))

We use the binomial theorem in Equations (9) and (10) to obtain $\Theta_{1}\left(P_{r}\left(C\right)\right))$ and $\Theta_{2}\left(P_{r}\left(C\right)\right))$, respectively.

(9) Θ1(Pr(C)))=∑mk=1N−1N−1mkMsNmk1−MsNN−mk−1+1−MsN−1−MsN=1−1−MsNN−1

(10) Θ2(Pr(C)))=∑mk=1N−1N−1mkMs−1Nmk1−MsNN−mk−1+1−MsN−1−MsN

$$={\left(\frac{M_{s}-1}{N}+1-\frac{M_{s}}{N}\right)}^{N-1}-{\left(1-\frac{M_{s}}{N}\right)}^{N-1}$$

Then by applying Equations (9) and (10) in Equation (8) we can obtain $P_{r}\left(C\right)$ as

(11) $$P_{r}\left(C\right)=1-{\left(1-\frac{M_{s}}{N}\right)}^{N-1}+{\left(\frac{M_{s}-1}{N}+1-\frac{M_{s}}{N}\right)}^{N-1}-{\left(1-\frac{M_{s}}{N}\right)}^{N-1}=1-{\left(1-\frac{1}{N}\right)}^{\left(N-1\right)}$$

For very high number of NB-IoT devices such that N→∞, we can further simplify Equation (11) as ($\lim_{N\to \infty }P_{r}\left(C\right)=1-e^{-1}$).

## 4. Average Power Consumption in Existing NB-IoT RAP

In this section, we evaluate the average power consumption by NB-IoT devices in the existing NB-IoT RAP. While the the choice of CE level for the first RAP attempt is random, for the subsequent reattempt it is not. Moreover, failure at any attempt can occur either due to collision or due to poor channel. This is due to the fact that unlike the proposed PE-RAP, the devices in existing NB-IoT RAP on failure do not check the channel again.

### 4.1. If the Device Starts RAP in CE0

In the existing NB-IoT RAP, the sequence of RAP reattempts as well as the number of designated reattempts in each of the CE levels are fixed. We consider that $L_{0}$ and $L_{1}$ are, respectively, the maximum number of attempts that the device performs in CE0 and CE1 before it can move to the next higher CE level for RAP reattempts. $\Delta P_{0}$, $\Delta P_{1}$ and $\Delta P_{2}$ delineate the power ramping at each attempt in CE0, CE1 and CE2, respectively. The power consumption of the device would not only depend upon the total number of attempts *n* but also on its relation with $L_{0}$ and $L_{1}$.

(a) For $n\leq L_{0}$:
  For the existing NB-IoT RAP, if the device starts RAP in CE0 and succeeds in CE0 itself (i.e., $n\leq L_{0}$), then the power consumed (${PE}_{0}\left[n\leq L_{0}\right]$) by this device can be expressed as
  (12) $$\begin{array}{c} P_{E_{0}}\left[n\leq L_{0}\right]=\sum_{u=1}^{n}R_{0}\left(P_{0}+\left(u-1\right)\Delta P_{0}\right) \end{array}$$
  In the existing NB-IoT RAP, the device first reattempts RAP in the same channel conditions (i.e., $q_{0}$ for CE0) for $L_{0}$ attempts before moving to the next higher CE levels if the failure persists. The probability that the device that starts RAP in CE0 and succeeds in CE0 itself in $n\leq L_{0}$ reattempts, $P_{r}\left(S_{00}\right)\left\{n\right\}$, can be expressed as
  (13) $$\begin{array}{cc} \; & P_{r}\left(S_{00}\right)\left\{n=1\right\}=1-P_{r}\left(C\right)) \end{array}$$
  (14) $$\begin{array}{cc} \; & P_{r}\left(S_{00}\right)\left\{1<n\leq L_{0}\right\}=P_{r}\left(C\right){\left(1-\left(1-P_{r}\left(C\right)\right)q_{0}\right)}^{\left(n-2\right)}\left(\left(1-P_{r}\left(C\right)\right)q_{0}\right) \end{array}$$
  It is noteworthy that unlike PE-RAP, in $P_{r}\left(S_{00}\right)\left\{1<n\leq L_{0}\right\}$ evaluations, both probabilities due to collision $P_{r}\left(C\right)$ and channel condition $q_{0}$ are considered. At any RAP reattempt the failure could have occurred due to collision or due to poor channel conditions. However, for the selection of its first attempt, the device measured RSRP and hence for $P_{r}\left(S_{00}\right)\left\{n=1\right\}$ the failure is considered only due to collision. The probability that a device that starts RAP in CE0 but is not able to succeed for the designated $L_{0}$ reattempts in CE0 can be expressed as
  (15) $$\begin{array}{c} P_{r}\left(F_{00}\right)=P_{r}\left(C\right){\left(1-\left(1-P_{r}\left(C\right)\right)q_{0}\right)}^{\left(L_{0}-1\right)} \end{array}$$
  Subsequently, if the RAP failure persists then the device moves on to CE1 for RAP reattempts.
(b) For $L_{0}<n\leq L_{0}+L_{1}$:
  If the device starts RAP in CE0 and succeeds in $L_{0}<n\leq L_{0}+L_{1}$ attempts, then power consumed by this device can be expressed as
  (16) $$\begin{array}{cc} \; & P_{E_{0}}\left[L_{0}<n\leq L_{0}+L_{1}\right]=R_{0}P_{0}L_{0}+R_{0}\Delta P_{0}\frac{\left(L_{0}-1\right)\left(L_{0}\right)}{2}+\sum_{v=1}^{n-L_{0}}R_{1}\left(P_{1}+\left(v-1\right)\Delta P_{1}\right) \end{array}$$
  The probability that the device succeeds in CE1, having started in CE0, in $n=L_{0}+1,L_{0}+2\ldots L_{0}+L_{1}$ attempts can be expressed as in Equation (17).
  (17) $$P_{r}\left(S_{01}\right)\left\{L_{0}<n\leq L_{1}\right\}=P_{F_{00}}{\left(1-(\left(1-P_{r}\left(C\right)\right)\left(q_{0}+q_{1}\right))\right)}^{n-L_{0}-1}\left(\left(1-P_{r}\left(C\right)\right)\left(q_{0}+q_{1}\right)\right)$$
  We can express $P_{r}\left(F_{01}\right)$ as the probability that a device that starts RAP in CE0 but is not able to succeed for the designated $L_{0}$ reattempts in CE0 as well as the configured $L_{1}$ reattempts in CE1. It can be obtained as
  (18) $$\begin{array}{cc} P_{r}\left(F_{01}\right) & =P_{F_{00}}{\left(1-(\left(1-P_{r}\left(C\right)\right)\left(q_{0}+q_{1}\right))\right)}^{L_{1}} \end{array}$$
  The RAP failure in Equation (18) considers collision probability ($P_{r}\left(C\right)$) as well as channel probabilities $q_{0}$ & $q_{1}$. Finally the device tries reattempts in CE2.
(c) For $n>L_{0}+L_{1}$:
  For the device that starts RAP in CE0 but succeeds in $n>L_{0}+L_{1}$ attempts, the power consumed can be obtained as
  (19) $$\begin{array}{cc} P_{E_{0}}\left[n>L_{0}+L_{1}\right]= & R_{0}P_{0}L_{0}+R_{0}\Delta P_{0}\frac{\left(L_{0}-1\right)\left(L_{0}\right)}{2}+R_{1}P_{1}L_{1}+R_{1}\Delta P_{1}\frac{\left(L_{1}-1\right)\left(L_{1}\right)}{2}\\ \; & +\sum_{w=1}^{n-L_{0}-L1}R_{2}\left(P_{2}+\left(w-1\right)\Delta P_{2}\right) \end{array}$$
  As CE2 is the final CE level, the device after failure in the level has no choice but to reattempt in CE2 itself. Thus, the probability that the device succeeds in CE2 after having started in CE0 in $n=L_{0}+L_{1}+w$ attempts can be expressed as
  (20) $$\begin{array}{c} P_{r}\left(S_{02}\right)\left\{n>L_{0}+L_{1}\right\}=P_{F_{01}}P_{r}{\left(C\right)}^{\left(n-L_{0}-L1-1\right)}\left(1-P_{r}\left(C\right)\right) \end{array}$$
  The average power consumed by the device that manifests the very first attempt at RAP in CE0, unconditioned to *n*, can be expressed as
  (21) $$\begin{array}{cc} P_{{E_{0}}_{avg}}= & \sum_{n=1}^{L_{0}}P_{E_{0}}\left[n\leq L_{0}\right]P_{r}\left(S_{00}\right)\left\{n\right\}+\sum_{n=L_{0}+1}^{L_{0}+L_{1}}P_{E_{0}}\left[L_{0}<n\leq L_{0}+L_{1}\right]P_{r}\left(S_{01}\right)\left\{n\right\}\\ \; & +\sum_{n=L_{0}+L1+1}^{L_{0}+L_{1}+w}P_{E_{0}}\left[n>L_{0}+L_{1}\right]P_{r}\left(S_{02}\right)\left\{n\right\} \end{array}$$

### 4.2. If the Device Starts RAP in CE1

Let us consider $P_{E_{1}}\left[n\right]$ as the power consumed over *n* reattempts if the device starts RAP in CE1.

(a) For $n\leq L_{1}$:
  If the device starts RAP in CE1 and succeeds in *n* attempts (such that $n\leq L_{1}$) while power ramping is applied, then the power consumed (${PE}_{1}\left[n\leq L_{1}\right]$) by this device can be expressed as
  (22) $$\begin{array}{c} P_{E_{1}}\left[n\leq L_{1}\right]=\sum_{s=1}^{n}R_{1}\left(P_{1}+\left(s-1\right)\Delta P_{1}\right) \end{array}$$
  If the device starts RAP in CE1, then the probability that it succeeds in the first attempt can be expressed as $\left(P_{r}\left(S_{11}\right)\left\{n=1\right\}=1-P_{r}\left(C\right)\right)$. However, on failure, first it reattempts in CE1 itself for $L_{1}$ attempts and subsequently moves to CE2 if the failure persists. The probability that the device that starts RAP in CE1 and succeeds in CE1 itself in $n=2,3,\ldots L_{1}$ reattempts can be expressed as in Equation (23).
  (23) $$P_{r}\left(S_{11}\right)\left\{1<n\leq L_{1}\right\}=P_{r}\left(C\right)(1-{\left(\left(1-P_{r}\left(C\right)\right)q_{1})\right)}^{\left(n-2\right)}\left((\left(1-P_{r}\left(C\right)\right)q_{1})\right)$$
  The probability that a device that starts RAP in CE1 but does not succeeds for designated $L_{1}$ reattempts in CE1 can be expressed as
  (24) $$\begin{array}{c} P_{r}\left(F_{11}\right)\left\{n=L_{1}\right\}=P_{r}\left(C\right){\left(1-(1-P_{r}\left(C\right)q_{1})\right)}^{\left(L_{1}-1\right)} \end{array}$$
  Subsequently, the device that does not succeed in $L_{1}$ reattempts in CE1 moves to CE2.
(b) For $n>L_{1}$:
  The device that started RAP in CE1, that succeeded in *n* (such that $n>L_{1}$) attempts, and then that was power consumed by this device can be expressed as
  (25) $$\begin{array}{c} P_{E_{1}}\left[n>L1\right]=R_{1}P_{1}L_{1}+R_{1}\Delta P_{1}\frac{\left(L_{1}-1\right)\left(L_{1}\right)}{2}+\sum_{t=1}^{n-L_{1}}R_{2}\left(P_{2}+\left(t-1\right)\Delta P_{2}\right) \end{array}$$
  The probability that the device succeeds in CE2 after having started in CE1 in $n=L_{1}+t$ attempts can be expressed as
  (26) $$\begin{array}{cc} \; & P_{r}\left(S_{12}\right)\left\{n>L_{1}\right\}=P_{F_{11}}P_{r}{\left(C\right)}^{\left(n-L_{1}-1\right)}\left(1-P_{r}\left(C\right)\right) \end{array}$$
  The average power spent by a device in the existing system after having started in CE1, unconditioned to *n*, can be expressed as
  (27) $$\begin{array}{c} P_{{E_{1}}_{avg}}=\sum_{n=1}^{L_{1}}P_{E_{1}}\left[n\leq L_{1}\right]P_{r}\left(S_{11}\right)\left\{n\right\}+\sum_{n=L1+1}^{L_{1}+t}P_{E_{1}}\left[n>L_{1}\right]P_{r}\left(S_{12}\right)\left\{n\right\} \end{array}$$

### 4.3. If the Device Starts RAP in CE2

The average power spent by this device in *n* RAP attempts can be expressed as

(28) $$\begin{array}{cc} P_{E_{2}}\left[n\right] & =\sum_{r=1}^{n}R_{2}\left(P_{2}+\left(r-1\right)\Delta P_{2}\right) \end{array}$$

The average power spent by a device in existing NB-IoT RAP having started in CE2, unconditioned to *n*, can be expressed as

(29) $$\begin{array}{cc} P_{{E_{2}}_{avg}} & =\sum_{n=1}^{\infty }P_{E_{2}}\left[n\right]\left(P_{r}{\left(C\right)}^{\left(n-1\right)}\left(1-P_{r}\left(C\right)\right)\right) \end{array}$$

### 4.4. Average Power in Existing NB-IoT RAP

Finally, we can obtain the average power spent by a device in an existing NB-IoT RAP after having started in any of the CE levels using the aforesaid analysis as

(30) $$\begin{array}{cc} P_{E_{avg}} & =q_{0}P_{E_{0_{avg}}}+q_{1}P_{E_{1_{avg}}}+q_{2}P_{E_{2_{avg}}} \end{array}$$

## 5. Performance Evaluations

In this section we first present the numerical results based on our analysis of power consumption in PE-RAP and compare them to the existing system. Subsequently, we also validate our analysis through simulation results. The power consumed by the device in a CE level depends upon the number of configured repetitions. The network can configure the number of repetitions in a CE level based on channel conditions. Instead of considering only a single value, we perform analysis over a range of repetition values in all the three CE levels. We consider variations in the number of repetitions in CE0 as 2∼16, in CE1 as 4∼32 and in CE2 as 8∼64. The performance parameters are given in Table 3.

In massive connectivity, a large number of devices may simultaneously send transient and rapid session requests in a short time to the network [28]. The classical homogeneous/compound Poisson process is suitable for occasional and steady traffic arrival but can hardly describe the simultaneous burst [32,33]. It is expected that the 5G network would support over a million devices with frequent bursty traffic arrival. In such scenarios, in each time slot a large number of devices may attempt the transmission of the preambles. As a result, collisions may occur more frequently accumulating into a critical congestion. According to 3GPP, the Beta distribution emulates the case when umpteen devices try to synchronously access the network [34] and thus gives a better perception about bursty Machine Type Communications (MTC) traffic. To emulate massive connectivity in NB-IoT we can assume that several devices try to access at time t∈(0;T) while following a Beta distribution [25], with the probability density function as:

(31) $$\begin{array}{cc} f_{x}\left(x\right) & =\frac{x^{a-1}{\left(T-x\right)}^{b-1}}{{\left(T\right)}^{a+b-1}B\left(a,b\right)} \end{array}$$

where, a=3 and b=4[34]. B(a,b) represents Beta function $\int_{0}^{1}x^{a-1}{\left(1-x\right)}^{b-1}dx$.

The NPRACH design in NB-IoT manifests 48 subcarriers on an anchor that can be used as a preamble for RAP. Moreover, the basic allocation unit that can be assigned to any CE level comprises of 12 sub-carriers as explained in Section 2. We consider the same number of preambles for CE0 and CE1 valued at 12. We consider CE2 to be configured with the remainder out of 48 preambles. More preambles are considered in CE2 since in legacy network, the devices that fail in CE0 and CE1, ultimately try RAP attempt in CE2. To avoid high collisions in CE2 more preambles are allocated to CE2. The collided devices can jump from lower CE levels to higher level CEs in existing systems while they attempt to successfully transmit their preambles. The devices in PE-RAP can flexibly jump from one level to another based on RSRP measurements. However, the number of reattempts that the device can perform in each CE level is fixed. We consider the device to be able to attempt for the maximum of five times in each CE level after which it is considered as a failure. It is to be noted that each attempt in turn comprises of several repetitions. Each repetition is assumed to consume a unit power in level CE0. In CE1 the power consumption per repetition is doubled, while in CE2 it is considered to be thrice the power consumption in CE0. Here, we have chosen the aforesaid simple setting to represent the comparison of our proposal with the existing one. Note that this consideration can be changed according to the real world situation. However, since we highlight the comparison, the actual values when used in both the existing and proposed RAP would result in the same trends.

**Figure 4:**
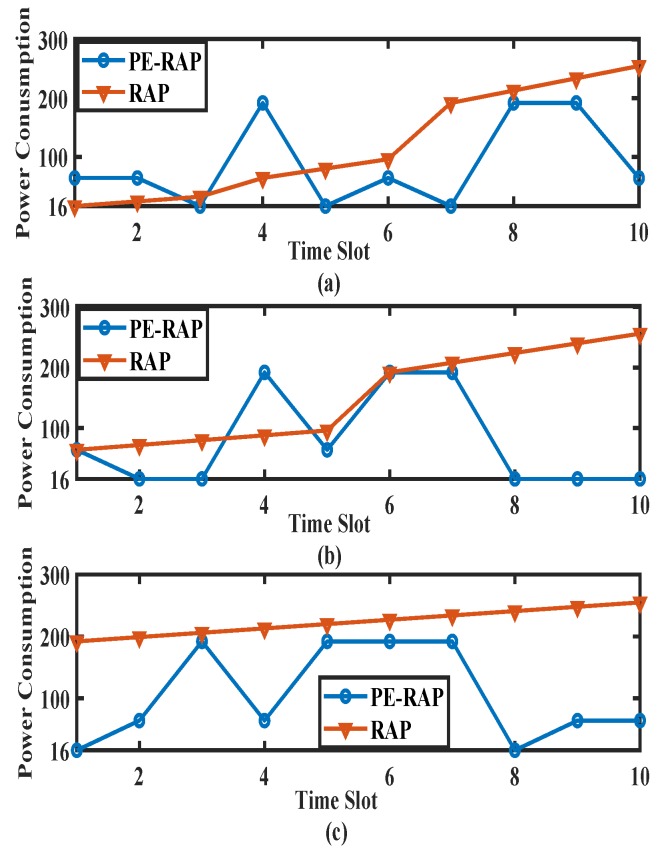
Snapshot of power consumption variations over several reattempts for proposed PE-RAP and existing NB-IoT RAP. (**a**) Device makes first RAP attempt in CE0; (**b**) Device makes first RAP attempt in CE1; (**c**) Device makes first RAP attempt in CE2.

Figure 4a–c shows the variation in a device’s power consumption for several RAP reattempts after having performed the first RAP attempt in CE0, CE1 and CE2, respectively. For the existing RAP, in Figure 4a the devices first try RAP in CE0 such that the transmission takes place at the lower power level with a lower number of repetitions. In Figure 4b,c, the device first attempts RAP in CE1 and CE2, respectively. Thus, the starting power level for the existing RAP is maximum for Figure 4c and minimum for Figure 4a. As the number of reattempts increases, the power consumption also increases in the existing system. For the proposed PE-RAP, the CE level is re-ascertained at each attempt. The number of repetitions and the power level per repetition assigned to each CE level for the proposed PE-RAP are different. Thus, the power consumed by the device in its attempt in CE0 would be different from its attempt in CE1 or CE2. It is notable that due to the random selection of the CE level by the NB-IoT device after an RAP failure, the power consumed may vary randomly over different trials and our proposed algorithm tries to adapt the same dynamics. Figure 4 clearly shows these dynamics. For an attempt the device may choose a lower CE0 level with less power consumption. In the next reattempt, it may select CE2, and it the subsequent reattempt CE1. This would result in variations as shown in Figure 4. It can be observed that at some instantaneous attempts the power level for the existing RAP is lower than that for the proposed PE-RAP while for some other attempts it is higher. Thus, it is important to consider the average power consumption of the devices as highlighted in Equations (2) and (3).

**Figure 5:**
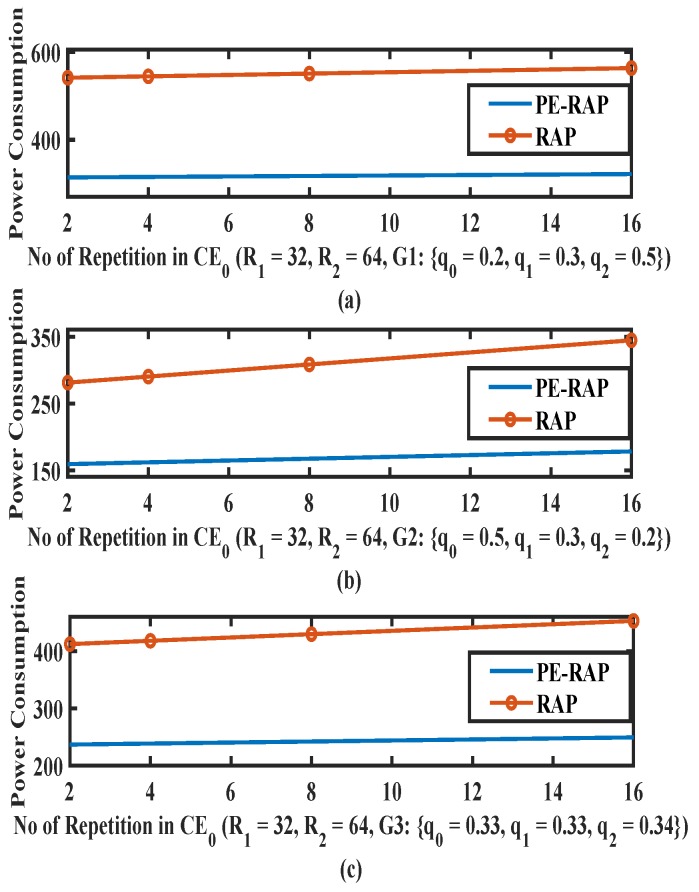
Average power consumption over varying repetitions per attempt in CE0 (CE = coverage enhancement) for proposed PE-RAP and existing NB-IoT RAP. (**a**) Low value of $q_{0}$; (**b**) High value of $q_{0}$; (**c**) Average value of $q_{0}$.

In Figure 5 we show the the average power consumption for existing RAP and proposed PE-RAP for a varying number of configured repetitions in CE0. The number of repetitions in CE1 and CE2 are fixed to 32 and 64, respectively. We assume that the device makes initial RAP attempt in CE0. In the proposed PE-RAP the device transits to different CE levels based on channel conditions. Since the power consumed in each CE level is different, it is important to consider the probability of selection of a particular CE level. As discussed before, $q_{0},\;q_{1}$ and $q_{2}$, respectively, represent the probabilities of channel conditions for CE level CE0, CE1 and CE2 while $q_{0}+q_{1}+q_{2}=1$. For our analysis we consider three cases:

1. Probability of selection of CE0 is high. To emulate this scenario we consider $\left\{q_{0},\;q_{1},\;q_{2}\right\}=\left\{0.5,\;0.3,\;0.2\right\}$.
2. CE0 manifests lower probability of selection. To consider this case we take $\left\{q_{0},\;q_{1},\;q_{2}\right\}=\left\{0.2,\;0.3,\;0.5\right\}$.
3. Probability of selection of CE0 is average such that $\left\{q_{0},\;q_{1},\;q_{2}\right\}=\left\{0.33,\;0.33,\;0.34\right\}$

As an example, if an NB-IoT device is indoors but on ground floor or a higher floor, then the measured RSRP value would be good. The probability $q_{0}$ would be higher than $q_{1}$ or $q_{2}$. However, if the device is indoors in the basement, then $q_{2}$ would be higher than $q_{0}$ or $q_{1}$. In the proposed PE-RAP, the device fails RAP due to collision and a reattempt is performed in a CE level based on the observed channel condition. In Figure 5a there is high probability that the device selects CE0 again after failure. The number of repetitions as well as the power level per repetition are small in CE0 . Thus, in Figure 5a the average power consumption for the proposed PE-RAP is almost around 40% less than the existing RAP for a different number of configured repetitions in CE0. In Figure 5b, even when the probability of selection of CE0 is low, power saving is substantial. This is because in the proposed PE-RAP the device can quickly re-select a higher CE level while avoiding the sequential stepping up resulting in early success of RAP. On the other hand, in the existing RAP, the power levels are ramped sequentially until the device is able to succeed.

**Figure 6:**
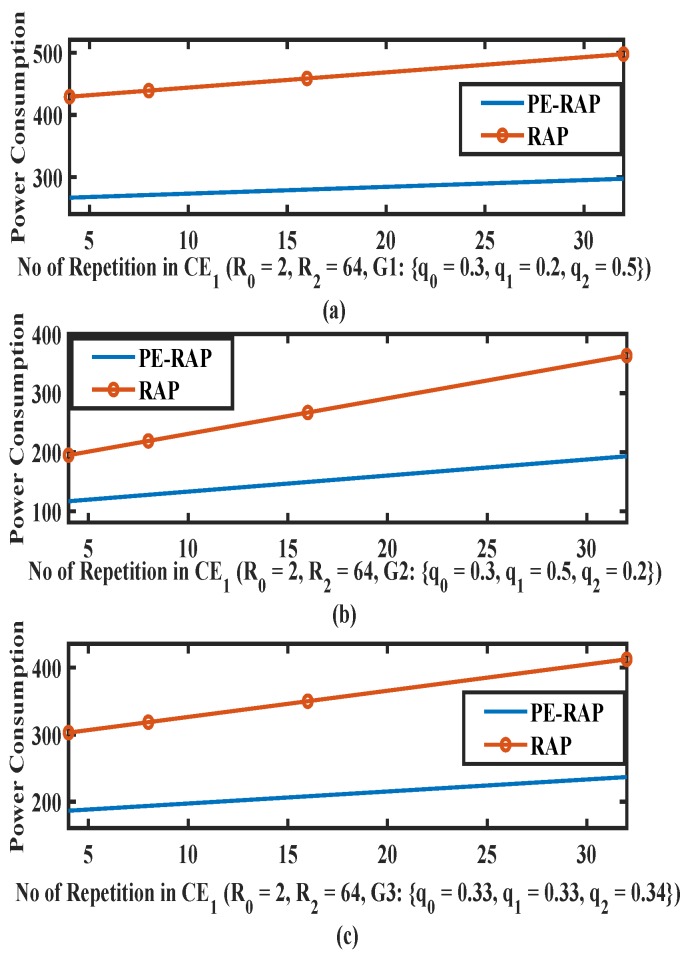
Average power consumption over varying repetitions per attempt in CE1 for proposed PE-RAP and existing NB-IoT RAP. (**a**) Low value of $q_{1}$; (**b**) High value of $q_{1}$; (**c**) Average value of $q_{1}$.

Figure 6 shows the average power consumption for an existing RAP and a proposed PE-RAP for a varying number of configured repetitions in level CE1. Here also we consider three cases: (i) Probability of selection of CE1 is high such that $\left\{q_{0},\;q_{1},\;q_{2}\right\}=\left\{0.3,\;0.5,\;0.2\right\}$; (ii) CE1 manifests lower probability such that $\left\{q_{0},\;q_{1},\;q_{2}\right\}=\left\{0.3,\;0.2,\;0.5\right\}$; (iii) probability of selection of CE1 is average such that $\left\{q_{0},\;q_{1},\;q_{2}\right\}=\left\{0.33,\;0.33,\;0.34\right\}$. It can be observed from Figure 6 that the power consumption for the proposed PE-RAP is generally lower (almost half) than the existing RAP for different configurations of repetition values. Moreover, the proposed PE-RAP performs better for the higher configuration of repetition per attempt.

**Figure 7:**
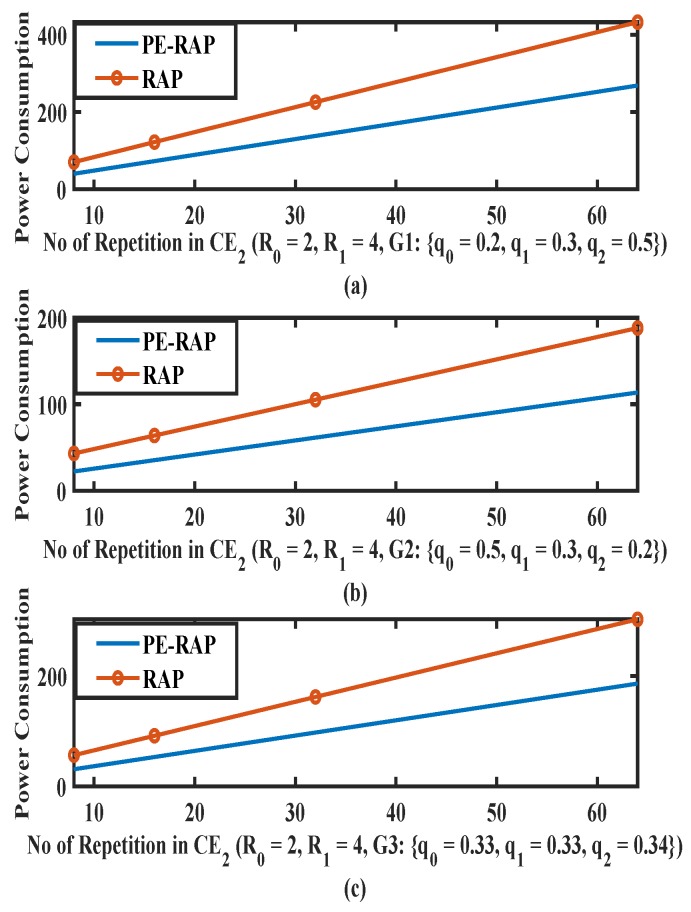
Average power consumption over varying repetitions per attempt in CE2 for proposed PE-RAP and existing NB-IoT RAP. (**a**) High value of $q_{2}$; (**b**) Low value of $q_{2}$; (**c**) Average value of $q_{2}$.

Figure 7 shows the power consumption with the varying number of repetitions for level CE2. The number of repetitions in CE0 and CE1 are kept fixed (CE0 repetition 2 and CE1 repetition 4). Similar cases as in Figure 5 and Figure 6 are considered, that is: (i) Probability of selection of CE2 is high such that $\left\{q_{0},\;q_{1},\;q_{2}\right\}=\left\{0.2,\;0.3,\;0.5\right\}$; (ii) CE2 manifests lower probability such that $\left\{q_{0},\;q_{1},\;q_{2}\right\}=\left\{0.5,\;0.3,\;0.2\right\}$; (iii) probability of selection of CE2 is average such that $\left\{q_{0},\;q_{1},\;q_{2}\right\}=\left\{0.33,\;0.33,\;0.34\right\}$. Initially the power consumed by the device is very low due to a lower number of repetitions. Since number of repetitions and power levels are high for CE2, the high channel selection probability manifests high power consumption (Figure 7a). Lower selection probability of $q_{2}$ means that the device selects CE0 and CE1 more often and thus the power consumption in Figure 7b is less than Figure 7a. The proposed PE-RAP performs better even in the highest CE level since the device has the flexibility to move from a higher CE level to a lower CE level for an RAP reattempt unlike the existing RAP resulting in the lower average power consumption.

A few common observations from Figure 5, Figure 6 and Figure 7: (i) As expected the power consumption increases as the number of repetitions increases for both the existing RAP and the proposed PE-RAP. (ii) As the number of repetitions increases the gap between the existing RAP and the PE-RAP increases. This is crucial since the fundamentals of the NB-IoT design are rooted in repetitions and the proposed PE-RAP manifests more power saving as the number of repetitions increases.

### Simulation Results

To validate the numerical results we performed the system level simulations. We used Matlab to implement our simulation scenario. Simulations were run 10,000 times. The simulation steps are explained as follow:

- We consider a single cell, where the active devices that are required to perform RAP are randomly distributed.
- A particular device that fails an RAP attempt checks the channel status for a reattempt and selects the CE level state based on channel condition.
- The CE level that the device selects consist of newly activated devices. It is considered that new device arrival follows Beta distribution, with a=3 and b=4.
- The selected CE level would also have already collided other devices that would perform RAP reattempt along with the device under consideration. Thus, the particular device competes for preamble with new activated devices as well as previously failed devices that happen to select this CE level after RSRP re-measurements.
- We first calculate the optimal value of the ACB factor by using the ratio M/N, where *M* is number of preambles and *N* is total number of devices at the start of every simulation slot. *N* comprises of the collided devices and the new arrivals.
- The device selects a random number between 0 and 1. The selected number is compared with the ACB factor. If the number is less than the ACB factor then the device does not transmit.
- The device selects the preamble for transmission in the specific time slot. We adopt the S-ALOHA transmission algorithm which divides time into consecutive slots. If two devices selects the same preamble in the same time slot then it is considered as collision. The device can only successfully transmit the preamble if it is different from other devices’.
- The collided devices again select the new CE level for transmission during the next time slot in PE-RAP and this process is repeated.
- For the selection of the CE level, the device generates a random number between 0 and 1 during each time slot. The number selected by the device is compared with $q_{0}$, $q_{1}$ and $q_{2}$. Based on the comparison, the CE level is selected. Thus, the choice of CE level for a reattempt would be different for all the devices. All the devices that attempt in one CE level in one slot might reattempt in a different CE level in the next slot.
- At the end of the simulation the number of attempts in each state is recorded. The simulation is repeated 10,000 times and results are averaged to obtain power consumption in every CE level.

**Figure 8:**
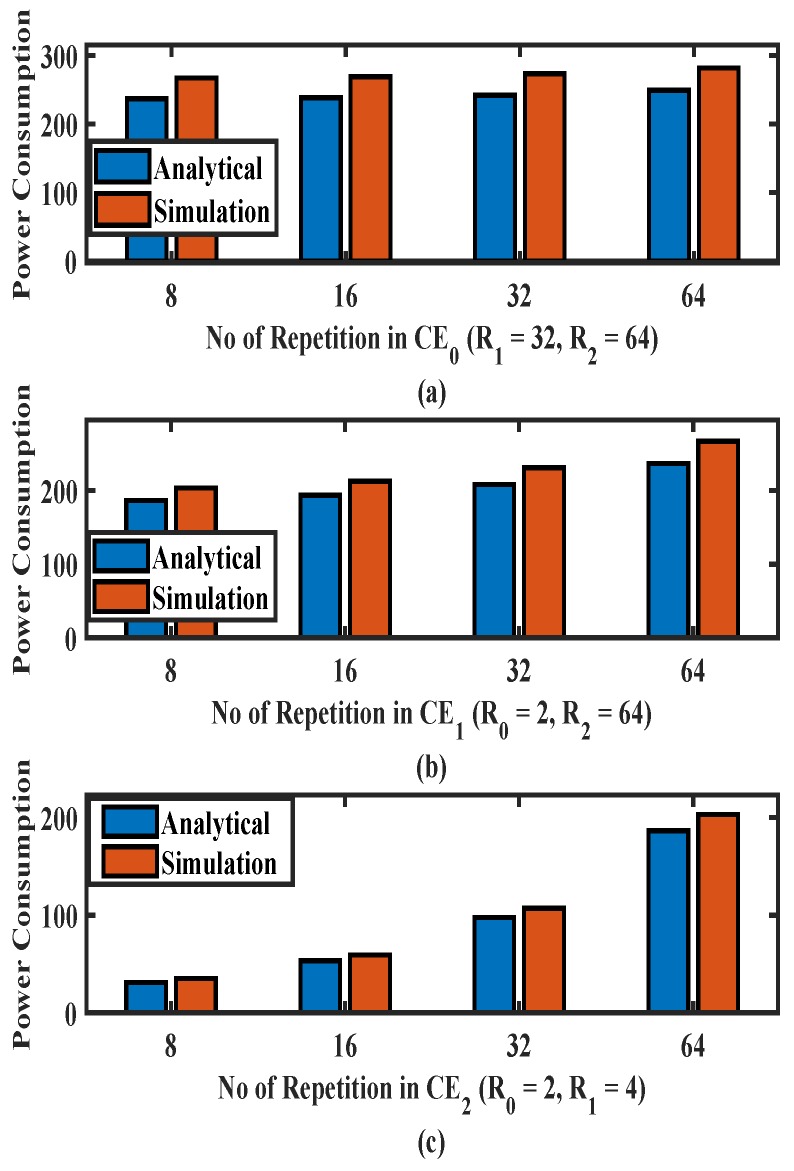
Power consumption comparison (analytical and simulation) for variations in repetitions (**a**) in CE0, (**b**) in CE1, (**c**) in CE2.

Figure 8 delineates the power consumption for PE-RAP obtained through the aforesaid simulations and it is compared to the results of our numerical analysis. In Figure 8a the number of repetitions in CE0 are varied from 2∼16 and the number of repetitions in CE1 and CE2 are fixed to 32 and 64, respectively. Figure 8b depicts the simulation and analytical results for a varying number of repetitions in CE1. The number of repetitions in CE0 and CE2 are fixed to 2 and 64, respectively. Similarly, in Figure 8c number of repetitions in CE2 are varied from 8 and 64, while the number of repetitions in CE0 and CE1 are fixed to 2 and 4, respectively. In Figure 8, as expected, the power consumption increases with the increase in the number of repetitions. It is observed from Figure 8a–c that the analytical results are comparable with the average of measured power consumption over all the simulation runs which validates our analysis.

**Figure 9:**
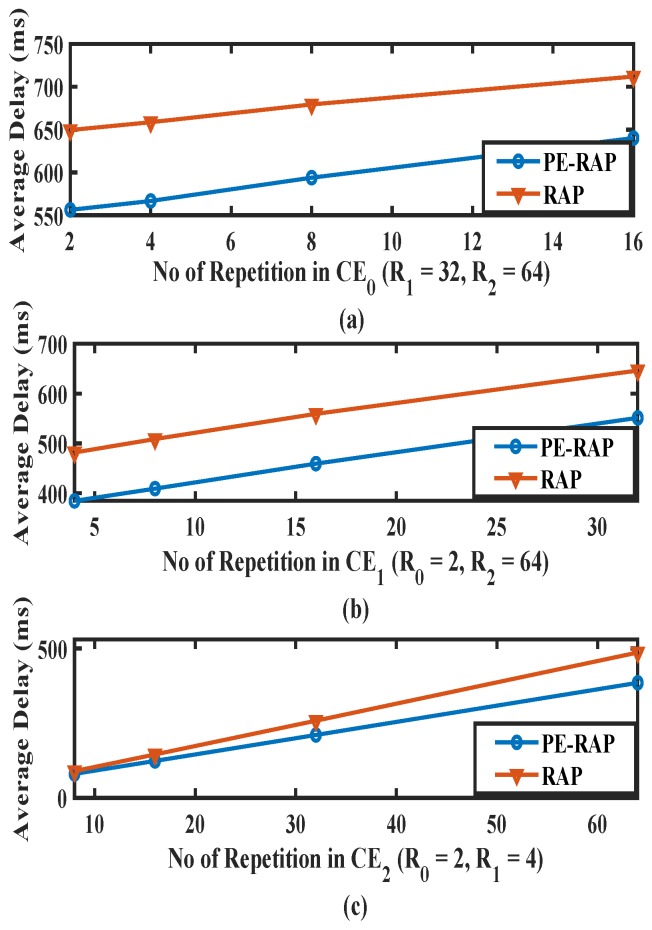
Average delay for different number of repetitions (**a**) in CE0, (**b**) in CE1, (**c**) in CE2 (proposed PE-RAP and existing RAP).

In the proposed PE-RAP, the device after collision can quickly select higher CE levels with more repetitions while reducing the number of failed attempts. More repetitions translate into more time in the each attempt. On the other hand, in existing RAP, more attempts, each with fewer repetitions, could be performed. More attempts each with less time would also add up to substantial delay. Thus, it becomes important to check the delay observed by devices in PE-RAP in comparison to existing RAP. We perform simulations for the average delay in PE-RAP and compare it to the existing RAP. We observe the time spent by devices in different CE levels before it is finally successful. Figure 9 shows the mean delay observed by a device for our proposed PE-RAP and existing RAP. The delay obtained by PE-RAP is less than the existing. This is because in existing RAP the device keeps trying in the initially chosen CE level for the configured number of reattempts before moving to the higher one. While in PE-RAP, the device ascertains and moves to the correct CE level sooner, which increases the chances of success in less time. As expected, the delay observed by the device increases with the increase in the number of configured repetitions.

**Figure 10:**
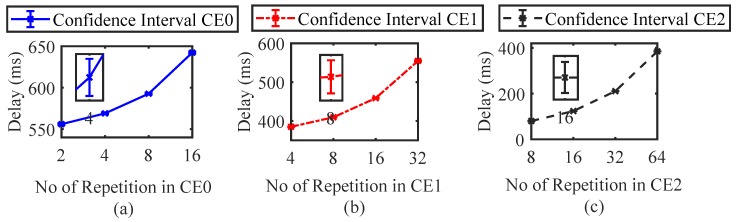
Confidence interval results for (**a**) CE0; (**b**) CE1; (**c**) CE2.

Figure 10 presents the confidence interval plot for different simulation results. We considered the confidence interval of 95%. We calculated the mean, standard deviation and margin of error. In Figure 10a, for CE0 when the number of repetitions is 4, the upper confidence level is 570.31 and lower confidence is 567.69. From Figure 9, it can be seen that for CE0 with 4 repetitions, the average of simulations is in the confidence interval range (567.69∼570.31). This verifies our simulations.

The PE-RAP advantages are based on the correct selection of the number of repetitions, the power level and the CE level. Incorrect selections would lead to more power consumption in power limited NB-IoT devices. The device measures RSRP for CE level selection more often in PE-RAP than the existing system. If the device measures RSRP incorrectly then it may select incorrect CE levels, more often resulting in repeated failures. For instance, if the device in CE2 keeps selecting CE0 incorrectly, it would never increment its power or repetitions and would never succeed. This would impact the performance and power consumption adversely. It would also increase the latency. On the other hand, if the channel condition is good, the device is expected to select CE0. However, if it measures RSRP incorrectly and selects CE2, it would suffer an unnecessary high power consumption, though success would be achieved faster. Furthermore, if the configured repetitions are small and the channel variations limited, then the device would have to perform RSRP measurements more frequently. Since our proposal is aimed at obtaining the effect of repetitions and reattempts on the average power consumption, the methodology for measurement of RSRP for the selection of CE levels is not in the paper and can be considered in future works. Another effect that can be considered is the allocation of preambles to different CE levels. If all users in a cell are located at the same distance with the same RSRP then they may select the same CE level which would increase the collision probability. In such a case more preambles should be allocated to this particular CE level. If the network can allocate preamble resources dynamically based on collision probability, the RAP reattempts and therefore impediments of several repetitions can be reduced.

## 6. Conclusions

Narrowband Internet of Things (NB-IoT) is expected to support a massive number of devices over a wider area of coverage. In NB-IoT, the concept of coverage enhancement (CE) level is included to support devices that operate at different path losses. CE levels are characterized by independent configurations for signal repetitions to achieve good signal quality at the desired coverage. The interaction among CE levels complicates the random access procedure (RAP) in NB-IoT. The RAP is expected to become even more challenging since NB-IoT devices would experience frequent collisions due to simultaneous access by an enormous number of devices. Enhancing power saving of the devices while performing RAP in such an environment is an important issue that is addressed in this article. We present power efficient RAP for reducing power consumption of the NB-IoT devices while they perform RAP in the congested environment where each RAP (re)attempt is configured with several repetitions. The proposed novel model avoids the indiscriminate power rampings and unnecessary CE level transitions. Analytical results of power saving gain are compared with the existing RAP and are also validated through simulation studies.

## Figures and Tables

**Table 1:** Related work on random access in NB-IoT.

Technology in Focus/Approach	Refs.	Work Summary	Objective(s)
NB-IoT Overview	[1,2,21,22]	Review of NB-IoT evolution, technologies, and issues	Provide overview of design for NB-IoT
RAP in NB-IoT	[7]	NB-IoT RAP modelled probabilistically using Markov chain	To calculate system throughput
RAP in NB-IoT	[14]	Trade off between repetition and RAP reattempts	Increasing the detection probability
RAP in NB-IoT	[9]	Analytical model for RAP considering three CE levels	Success probability and access delay estimations
RAP in NB-IoT	[23]	Joint optimization technique under a target delay constraint	Optimal configuration of NPRACH parameters Maximization of the access success probability
RAP in NB-IoT	[24]	Access reservation protocol with partial preamble transmission	Reducing collision probability
RAP in NB-IoT	[4]	Superimposed NPRACH preambles with multiple RAPs	Derive detection threshold
RAP in NB-IoT	[26]	Classification of back-off in massive NB-IoT connectivity	Capacity gain is estimated

**Table 2:** Parameters used for power efficient random access procedure PE-RAP analysis.

Parameter	Symbol	Description
Number of Repetitions	$R_{0},R_{1},R_{2}$	$R_{0},R_{1},R_{2}$, respectively to delineate the number of repetitions in CE0, CE1 and CE2
Probability of Device belonging to a CE level	$q_{0}$, $q_{1}$, $q_{2}$	$q_{0}$, $q_{1}$ and $q_{2}$ respectively gives the probability of channel condition for CE0, CE1 and CE2 such that $q_{0}$+ $q_{1}$ +$q_{2}$ = 1
Number of Devices	*N*	Number of NB-IoT devices
Preambles/Subcarriers	$M_{s}$	Number of subcarriers (preambles)
Collision Probability	$P_{r}\left(C\right)$	Probability of collision of a device
ACB Factor	ρ	Access Class Barring (ACB) factor transmitted by network
Number of Devices Passes ACB check	$m_{k}$	Devices Passes ACB Check and transmit during this time slot
Arrival	B(a, b)	Beta function with parameters a & b
Power Level	$P_{0},P_{1}$, $P_{2}$	Power levels of CE0, CE1 and CE2
Number of attempts	$L_{0}$, $L_{1}$	Maximum number of attempts that a device performs in CE0 and CE1
Power Consumed	$P_{r}\left\{n\right\}$	Power consumed by device after *n* attempts
Probability of RAP	$P_{avg}\left[n\right]$	Probability of an RAP being a success in n attempts
Average Power Spent	$P_{{E_{0}}_{avg}}$, $P_{{E_{1}}_{avg}}$, $P_{{E_{2}}_{avg}}$	Average power spent in CE0, CE1 and CE2

**Table 3:** Performance parameters.

Parameter	Value
No. of repetition in CE0 ($R_{0}$)	4∼16
No. of repetition in CE1 ($R_{1}$)	4∼32
No. of repetition in CE2 ($R_{2}$)	8∼64
Power Consumption per Repetition CE0 ($P_{0}$)	1 dBm
Power Consumption per Repetition CE1 ($P_{1}$)	2 dBm
Power Consumption per Repetition CE2 ($P_{2}$)	3 dBm
Arrival Rate [a, b]	[34]
No. of Preambles in CE0	12
No. of Preambles in CE1	12
No. of Preambles in CE2	24
